# The impact of primary sclerosing cholangitis or inflammatory bowel disease on cholangiocarcinoma phenotype, therapy, and survival

**DOI:** 10.1002/jgh3.12405

**Published:** 2020-08-11

**Authors:** Daljeet Chahal, Chris Shamatutu, Bill Salh, Janine Davies

**Affiliations:** ^1^ Division of Gastroenterology University of British Columbia Vancouver British Columbia Canada; ^2^ Department of Medicine University of British Columbia Vancouver British Columbia Canada; ^3^ Division of Medical Oncology BC Cancer Vancouver British Columbia Canada

**Keywords:** cancer, cholangiocarcinoma, liver transplant, primary sclerosing cholangitis

## Abstract

**Background and Aim:**

Primary sclerosing cholangitis (PSC), with or without inflammatory bowel disease (IBD), confers the risk of cholangiocarcinoma. Isolated IBD may be an independent risk factor for cholangiocarcinoma. We sought to compare cholangiocarcinoma phenotype and outcomes between patients with PSC, IBD, and neither.

**Methods:**

Patients with malignancy were separated into cohorts by the presence of PSC and IBD. Data regarding demographics, clinical presentation, therapeutic regimens, and survival were collected. Statistical analysis was carried out using GraphPad and R‐Studio.

**Results:**

Of 946 patients, 22 had PSC, and 18 had isolated IBD. PSC and IBD patients were younger than controls (*P* < 0.001, *P* = 0.01). Cholangiocarcinoma prevalence was estimated at 0.01% for IBD patients, 0.6% for PSC patients, and 0.002% for all other patients. All cohorts most often presented at stage 4. PSC patients presented more often at stage 3 (*P* = 0.04) and with perihilar disease (*P* = 0.001). Patients with PSC or IBD received less chemotherapy (*P* = 0.004, 0.01). Median overall survivals were 15 months (PSC), 11 months (IBD), and 10 months (controls) (*P* = 0.79). Patients with intrahepatic tumors had longer survival (*P* < 0.001). Curative intent resection improved survival in all cohorts (*P* < 0.001). Multivariate regression identified resection as a predictor of improved survival. Extrahepatic, perihilar, gallbladder, and unspecified biliary tumors were predictors of death.

**Conclusions:**

Cholangiocarcinoma presents at a late stage and portends dismal survival regardless of PSC or IBD status. Survival was dependent on tumor location and surgical resection. These data suggest that efforts should focus on developing protocols that are able to detect and treat cholangiocarcinoma in high‐risk populations (PSC) at an early stage.

## Graphical abstract

We sought to compare cholangiocarcinoma phenotype and outcomes between patients with primary sclerosing cholangitis (PSC), inflammatory bowel disease (IBD), and neither. We confirm that cholangiocarcinoma presents at a late stage and portends dismal survival regardless of PSC or IBD status. Survival was dependent on tumor location and surgical resection. These data suggest that efforts should focus on developing protocols able to detect and treat cholangiocarcinoma in high‐risk populations at an early stage.

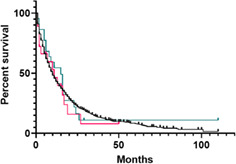

## Introduction

Cholangiocarcinoma (CCA) is the second most common primary hepatic malignancy and is further classified anatomically as intrahepatic (iCCA), perihilar (pCCA), or distal (dCCA).^1^ pCCA and dCCA are occasionally classified together as extrahepatic (eCCA). These subtypes differ in pathologic and mutational phenotypes, as well as clinical presentation, but all generally present at a late stage and have a dismal prognosis.^2^ The majority of CCA arises in patients without predisposing factors,^3^ in the seventh decade of life, and with a slight male predominance.^4^

Primary sclerosing cholangitis (PSC) is a chronic cholestatic disease, which results in progressive fibrosis of bile ducts.^5^ PSC confers a significantly increased lifetime risk of developing CCA and is responsible for a third of all‐cause mortality in these patients.^6^ Only 10% of all CCA can be attributed to PSC.^1^ These patients are young, with a strong male predominance.^6^ CCA arising from PSC may have a distinct morphomolecular phenotype irrespective of anatomical location.^7^ Despite improved outcomes associated with active CCA screening in PSC,^8^ guideline recommendations remain controversial due to a lack of therapeutic options.^6^ Finally, concurrent inflammatory bowel disease (IBD) in those with PSC confers additional CCA risk.^9^, ^10^ Several studies suggest that IBD may be an independent risk factor for CCA even in the absence of PSC,^11^, ^12^, ^13^ but conclusive evidence is lacking.

There is a paucity of studies directly comparing CCA in patients with PSC to those without PSC.^14^ There are no studies comparing such cohorts to those patients with IBD but no PSC who develop CCA. Given the distinct clinical profiles of patients with CCA (those without PSC, those with PSC, and those with IBD in the absence of PSC), we sought to compare disease behavior, management, and outcomes between these groups.

## Methods

### *Ethics and data*

The study protocol was reviewed and approved by the University of British Columbia/British Columbia Cancer Agency Research Ethics Board. The BC Cancer clinical and pharmacy databases were queried for all patients who were referred with a new diagnosis of biliary tract malignancy from 1 August 2009 to 1 December 2019. Data immediately available from the query included age, gender, tumor site, clinical stage, pathologic stage, overall stage, Eastern Cooperative Oncology Group (ECOG) performance status, surgical intent, surgical description, chemotherapy cycles, date of diagnosis, date of last contact, and date of death. From the BC Cancer database, as well as the Vancouver General Hospital electronic records, data regarding diagnosis of PSC, diagnosis of IBD, and liver transplantation were manually extracted. For those patients who had a diagnosis of IBD, IBD type (ulcerative colitis [UC] or Crohn's disease [CD]), duration of IBD, and history of bowel resection were further manually extracted from both data sources.

### *Disease definitions*

Clinical, pathologic, and overall disease stages specific to CCA subtype were defined in accordance with the American Joint Committee on Cancer (AJCC) staging system, 8th edition.^15^ Tumor locations were also defined in accordance with AJCC 8th edition. Gallbladder tumors were those originating in and isolated to the gallbladder. Extrahepatic tumors were defined as those distal to the confluence of the right and left hepatic ducts. Perihilar tumors in this study were defined as those involving or extending from the hepatic duct confluence proximally to second‐order bile ducts. Intrahepatic tumors were those found proximal to second‐order bile ducts (i.e. no involvement of first‐order bile ducts). The specifics of first‐ and second‐order bile duct anatomy may be found elsewhere.^16^ Overlapping biliary tumors were those that involved two or more anatomical regions. Biliary tumors not otherwise specified (NOS) were those where location was not documented.

### *Statistical analyses*

Patients were separated into cohorts by diagnosis. Cohorts included those with PSC (with or without IBD) (PSC+ | IBD±), those with IBD alone (PSC− | IBD+), and a control cohort consisting of those without PSC or IBD (PSC− | IBD−). Sample means were calculated for continuous variables, and categorical variables were tallied. A two‐tailed t‐test was used to compare continuous variables, and a two‐tailed z‐test was used to compare proportions of categorical variables. Diagnosis date, date of last contact, and date of death were used to construct Kaplan–Meier survival curves. Patients were censored if they were lost to follow‐up at the date of last contact. Log‐rank test was used to compare survival differences between cohorts. Univariate Cox hazard regression was performed on independent variables. Significant variables from the univariate analysis were used to perform a multivariate Cox hazard regression. Tests were considered significant if they had a *P*‐value less than 0.05.

This study was approved by the UBC Clinical Research Ethics Board.

## Results

### *Demographics*

A total of 946 patients were identified (Table 1). There were 4 patients with PSC alone, 18 with PSC and IBD, and 18 with IBD but no PSC (PSC− | IBD+). For the sake of analysis, all patients with PSC (with or without IBD) were grouped into one cohort (PSC+ | IBD±) (*n* = 22). The remaining 906 patients without diagnoses of PSC or IBD were used as the control cohort (PSC− | IBD−) (Table 1).

**Table 1:** Patient demographics

	PSC− | IBD+ (*n*, %)	PSC+ | IBD± (*n*, %)	PSC− | IBD−(controls) (*n*, %)	PSC− | IBD+ *versus* controls	PSC+ | IBD± *versus* controls
Total	18	22	906		
Age (mean)	62	46	68	0.007	<0.001
Gender (male)	5 (27.8)	16 (72.7)	428 (47.2)	0.1	0.02
Performance					
ECOG 0	1 (5.6)	3 (13.6)	62 (6.9)	0.83	0.22
ECOG 1	4 (22.2)	3 (13.6)	197 (21.7)	0.96	0.36
ECOG 2	4 (22.2)	3 (13.6)	151 (16.7)	0.53	0.40
ECOG 3	4 (22.2)	2 (9.1)	116 (12.8)	0.24	0.50
ECOG 4	1 (5.6)	1 (4.6)	48 (5.3)	0.96	0.88
Unknown	4 (22.2)	10 (45.5)	332 (36.6)		
Stage (diagnosis)					
I	0 (0.0)	1 (4.6)	54 (6.0)	0.29	0.78
II	4 (22.2)	3 (13.6)	127 (14.0)	0.32	0.96
III	0 (0.0)	4 (18.2)	62 (6.8)	0.25	0.04
IV	6 (33.3)	6 (27.3)	354 (39.1)	0.62	0.26
Unknown	8 (44.4)	8 (36.4)	309 (34.1)		
Tumor site					
Extrahep.	3 (16.7)	4 (18.2)	235 (25.9)	0.37	0.41
Perihilar	3 (16.7)	11 (50.0)	187 (20.6)	0.68	<0.001
Intrahep.	2 (11.1)	3 (13.6)	50 (5.5)	0.31	0.10
Gallblad.	6 (33.3)	3 (13.6)	256 (28.3)	0.64	0.13
Overlap	0 (0.0)	0 (0.0)	7 (0.8)	0.71	0.68
Bil. NOS	1 (5.6)	2 (9.1)	44 (4.9)	0.89	0.37
Ampulla	3 (16.7)	0 (0.0)	127 (14.0)	0.75	0.06
Chemotherapy					
None	17 (94.4)	21 (95.5)	595 (65.7)	0.01	<0.01
1 line	1 (5.6)	1 (4.6)	311 (34.3)	0.01	<0.01
2 lines	0 (0.0)	0 (0.0)	88 (9.7)	0.16	0.12
3 lines	0 (0.0)	0 (0.0)	25 (2.8)	0.47	0.43
Radiation					
None	17 (94.4)	20 (90.9)	787 (86.9)	0.34	0.58
1 cycle	1 (5.6)	2 (9.1)	119 (13.1)	0.34	0.58
2 cycles	0 (0.0)	2 (9.1)	45 (5.0)	0.33	0.38
3 cycles	0 (0.0)	2 (9.1)	45 (5.0)	0.33	0.38
4 cycles	0 (0.0)	0 (0.0)	15 (1.7)	0.58	0.54
5 cycles	0 (0.0)	0 (0.0)	15 (1.7)	0.58	0.54
Surgery					
None	10 (55.6)	13 (59.1)	517 (57.1)	0.90	0.85
Curative	6 (33.3)	9 (40.9)	323 (35.7)	0.84	0.61
Palliative	2 (11.1)	0 (0.0)	66 (7.3)	0.54	0.19
Liver transplant	0 (0.0)	5 (22.7)	4 (0.4)	0.78	<0.001

ECOG, Eastern Cooperative Oncology Group; IBD, inflammatory bowel disease; PSC, primary sclerosing cholangitis.

PSC+ | IBD± patients were significantly younger than controls (46 *vs* 68 years, *P* < 0.001), as were PSC− | IBD+ patients (62 *vs* 68, *P* = 0.01). Patients with PSC+ | IBD± were more likely to be male compared to controls (72.73 *vs* 47.24%, *P* = 0.02). There was no statistical difference in performance status (ECOG score) between the different cohorts at the time of diagnosis. Median time from diagnosis of PSC to diagnosis of CCA was 5.0 years.

### *IBD* *phenotype*

In PSC− | IBD+ patients, 10 (55.56%) had UC, and 8 (44.44%) had CD. In PSC+ | IBD± patients, 16 (88.89%) had UC, and 2 (11.11%) had CD (*P* = 0.03). There was no statistical difference in average duration of IBD in those with PSC compared to those without (19.88 years *vs* 26.85 years, *P* = 0.12). Five patients with PSC‐IBD had undergone colonic resection (two hemicolectomies, three total colectomies) compared to three patients with only IBD (two hemicolectomies, one total colectomy) (*P* = 0.42).

### *CCA* *prevalence estimation*

Simple mathematic estimates were calculated to compare the incidence of CCA between cohorts. We used conservative estimates of a Canadian population of approximately 35 million, IBD prevalence of 0.5% (1 per 200 persons; 500 per 100 000 persons),^17^ and PSC prevalence of 0.01% (1 per 10 000 persons; 10 per 100 000 persons).^18^, ^19^ Using the total number of patients in each cohort as an estimate of CCA prevalence within each of these populations, we calculated CCA estimates of 0.01% for IBD patients, 0.6% for PSC patients, and 0.002% for patients without IBD or PSC. These estimates suggest a higher frequency of CCA in both IBD patients (*P* < 0.001) and PSC patients (*P* < 0.001) compared to controls.

### *Cholangiocarcinoma phenotype*

When examining overall stage, all cohorts most often presented at stage 4 with distant metastases (PSC+ | IBD± 27.27%, PSC− | IBD+ 33.33%, controls 39.07%, *P* = 0.62, 0.26) (Table 1). Very few patients presented with stage 1 disease (PSC+ | IBD± 4.55%, PSC− | IBD+ 0.00%, Controls 5.96%, *P* = 0.29, 0.78). PSC+ | IBD± patients were more likely to present at stage 3 (local spread) than controls (18.18 *vs* 6.84%, *P* = 0.04). There were no significant differences when comparing individual clinical tumor node metastasis (cTNM) or pathologic TNM (pTNM) stage between cohorts (data not shown).

PSC+ | IBD± patients were more likely to present with perihilar disease than controls (50.00 *vs* 20.64%, *P* < 0.001), but otherwise, location of the primary site was not different. There was no statistical difference in the proportion of patients presenting with extrahepatic metastases. In addition, there was no difference in patients presenting with gallbladder malignancy. Intrahepatic CCA more often presented with stage 3 disease (data not shown), in both the PSC and control cohorts. Disease stage was most frequently absent for intrahepatic tumors.

### *Chemotherapy, radiation, and surgical resection*

Most PSC+ | IBD± patients (95.45%) and PSC− | IBD+ patients (94.44%) did not receive chemotherapy compared to 65.67% of controls (*P* = 0.003 and *P* = 0.01, respectively, Table 1). No patients with PSC or IBD advanced to second‐line chemotherapy, whereas 9.71% of controls did.

There were no differences in the rates of radiation therapy or surgical resection. While resection with curative intent was pursued more often than palliative surgery in all groups, there were no statistical differences between cohorts.

### *Transplantation and survival analyses*

Nine patients underwent orthotopic liver transplantation (OLT) at some point in their disease course (not necessarily for CCA). No PSC− | IBD+ patients underwent OLT, compared to four controls (0.00 *vs* 0.44%, *P* = 0.78). Five PSC+ | IBD± patients underwent OLT, which was significantly higher than control patients (22.72 *vs* 0.44%, *P* < 0.001); all of these had a concurrent diagnosis of IBD. One PSC+ | IBD± patient who underwent OLT required a second transplant. Transplant indication for PSC+ | IBD± patients included PSC with liver decompensation (*n* = 2), recurrence of PSC in the allograft (*n* = 1), CCA (*n* = 1), and unknown (*n* = 1). One control patient who underwent OLT required both a second and third transplant; otherwise, indications for liver transplantation could not be obtained (*n* = 3).

**Figure 1:**
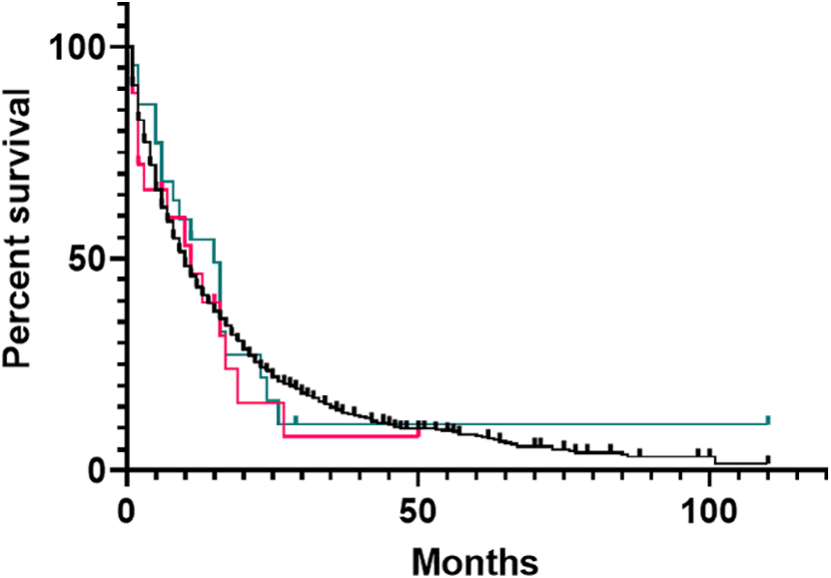
Overall survival of primary sclerosing cholangitis (PSC)+ | inflammatory bowel disease (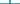) (IBD)±, (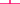) PSC− | IBD+, and (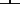) control cohorts (*P* = 0.79).

**Figure 2:**
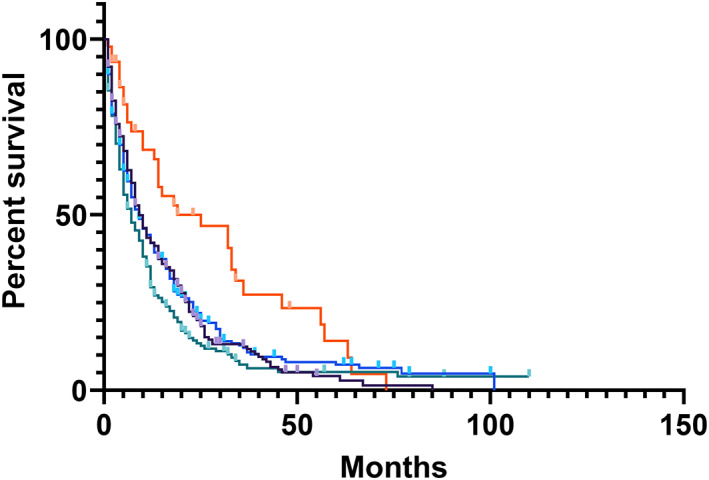
Overall survival by location of disease. Cholangiocarcinoma (CCA) classified as “intrahepatic” had increased survival relative to CCA classified as “extrahepatic,” “Perihilar,” or “gallbladder” (*P* < 0.001). Extrahepatic (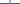), Gallbladder (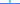), Perihilar (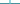), Intrahepatic (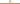).

**Figure 3:**
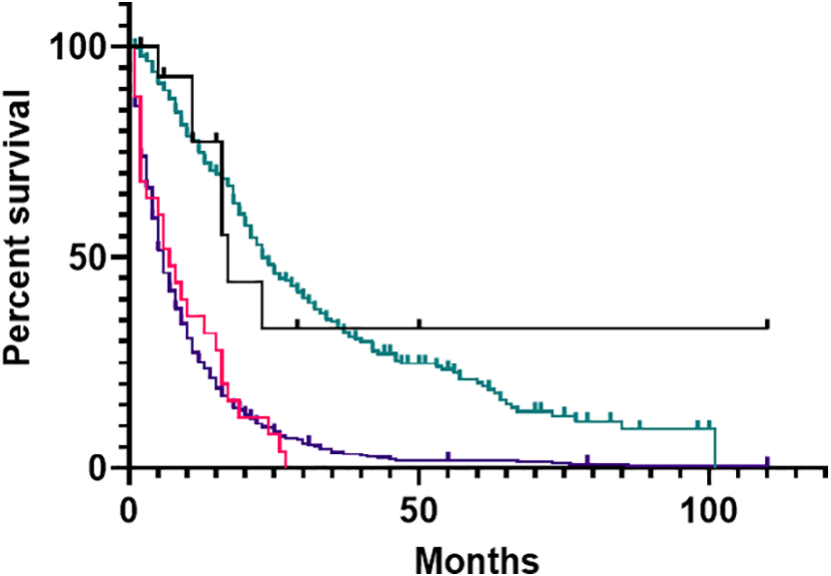
Overall survival in those who received curative intent surgery *versus* those who did not. In this analysis of primary sclerosing cholangitis (PSC)+ | inflammatory bowel disease (IBD)± and PSC− | IBD+ cohorts were combined into one larger cohort of 40 patients. All cohorts demonstrate increased survival with curative resection (*P* < 0.001). IBD/PSC Surgery (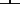), IBD/PSC No Surgery (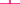), Control Surgery (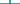), Control No Surgery (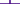)

Median overall survival (OS) for PSC+ | IBD± patients was 15 months, for PSC− | IBD+ patients 11 months, and for controls 10 months (Fig. 1, *P* = 0.79). Patients with intrahepatic tumors had longer survival (19 months) compared to patients with gallbladder (9 months), extrahepatic (9 months), and perihilar (7 months) tumors (*P* < 0.001) (Fig. 2). Patients in each cohort who underwent curative intent surgical resection had increased survival compared to those who did not (*P* < 0.001, Fig. 3), with survivals of 17 *versus* 8 months for PSC+ | IBD± patients, 16 months *versus* 5 months for PSC− | IBD+ patients, and 23 months *versus* 8 months for controls.

### *Cox proportional hazard regressions*

In univariate analyses (Table 2), curative intent surgical resection was associated with a decreased risk of death (hazard ratio [HR] 0.28, 95% confidence interval [CI] 0.24–0.33, *P* < 0.001). Liver transplantation also trended toward decreased risk of death but did not reach significance (HR 0.39, 95% CI 0.15–1.05, *P* = 0.06). There was an increased risk of death for extrahepatic (HR 2.16, 95% CI 1.67–2.78, *P* < 0.001), perihilar (HR 2.62, 95% CI 1.01–3.40, *P* < 0.001), and gallbladder (HR 2.13, 95% CI 1.66–2.74, *P* < 0.001) tumors, but no difference for intrahepatic tumors (HR 1.38, 95% CI 0.95–2.01, *P* = 0.09). Biliary tumors of unspecified or overlapping location, palliative surgery, increasing age, increasing overall disease stage, and increasing ECOG score were all also associated with increased risk of death. Gender, PSC, and IBD were not associated with increased risk of death.

**Table 2:** Univariate regression

Variable	HR	95% CI L	95% CI H	*P*‐Value
Curative surgery	0.28	0.24	0.33	<0.001
Liver transplant	0.39	0.15	1.05	0.06
Palliative surgery	1.42	1.10	1.84	<0.001
Stage				
I	NA	NA	NA	NA
II	1.21	0.78	1.86	0.39
III	2.03	1.29	3.20	<0.001
IV	5.62	3.80	8.32	<0.001
Unknown	2.98	2.02	4.41	<0.001
Tumor site				
Bil NOS	5.15	3.57	7.43	<0.001
Overlap	5.02	2.31	10.90	<0.001
Extrahep.	2.16	1.67	2.78	<0.001
Gallblad.	2.13	1.66	2.74	<0.001
Perihilar	2.62	1.02	3.40	<0.001
Intrahep.	1.38	0.95	2.01	0.09
PSC	0.86	0.54	1.37	0.51
IBD	0.94	0.64	1.37	0.74
IBD Leng.	0.99	0.97	1.02	0.71
IBD Rsxn.	0.57	0.23	1.40	0.22
Age	1.01	1.01	1.02	<0.001
Gender	0.98	0.86	1.14	0.86
Grade				
1	NA	NA	NA	NA
2	1.35	0.94	1.95	0.11
3	1.88	1.30	2.73	<0.001
4	1.39	0.43	4.50	0.59
Unknown	2.54	1.80	2.58	<0.001
Performance				
ECOG 0	NA	NA	NA	NA
ECOG 1	1.24	0.88	1.74	0.21
ECOG 2	2.41	1.71	3.39	<0.001
ECOG 3	4.31	3.02	6.14	<0.001
ECOG 4	8.68	5.71	13.18	<0.001
Unknown	1.78	1.29	2.45	<0.001

CI, confidence interval; ECOG, Eastern Cooperative Oncology Group; HR, hazard ratio; IBD, inflammatory bowel disease; NA, not available; NOS, not otherwise specified; PSC, primary sclerosing cholangitis.

Significant predictors of the univariate analysis were used to construct a multivariate Cox proportional hazard regression model (Table 3). Curative intent surgical resection remained a significant predictor of the decreased risk of death (HR 0.41, 95% CI 0.33–0.50, *P* < 0.001). Extrahepatic (HR 1.81, 95% CI 1.38–2.38, *P* < 0.001), perihilar (HR 1.76, 95% CI 1.33–2.33, *P* < 0.001), gallbladder (HR 2.06, 95% CI 1.58–2.68, *P* < 0.001), and biliary tumors of unspecified or overlapping location remained significant predictors of increased risk of death. Increasing overall disease stage and increasing ECOG score remained significant predictors of death, whereas palliative surgery and age did not reach significance in the multivariate model (Table 3).

**Table 3:** Multivariate regression

Variable	HR	95% CI L	95% CI H	*P*‐Value
Curative surgery	0.41	0.33	0.50	<0.001
Palliative surgery	0.78	0.59	1.00	0.05
Stage				
I				
II	1.43	0.92	2.24	0.11
III	2.10	1.31	3.39	<0.001
IV	3.24	2.14	4.92	<0.001
Unknown	1.93	1.27	2.91	<0.001
Tumor site				
Bil NOS	2.85	1.93	4.21	<0.001
Overlap	2.50	1.14	5.48	0.02
Extrahep.	1.81	1.38	2.38	<0.001
Gallblad.	2.06	1.58	2.68	<0.001
Perihilar	1.76	1.33	2.33	<0.001
Intrahep.	1.15	0.77	1.72	0.49
Age	1.01	0.99	1.01	0.12
Grade				
1				
2	1.29	0.89	1.87	0.18
3	1.54	1.05	2.25	0.03
4	2.09	0.63	6.92	0.23
Unknown	1.40	0.99	2.00	0.06
Performance				
ECOG 0				
ECOG 1	1.27	0.90	1.79	0.18
ECOG 2	2.20	1.55	3.11	<0.001
ECOG 3	4.21	2.93	6.06	<0.001
ECOG 4	8.56	5.55	13.20	<0.001
Unknown	1.68	1.21	2.33	<0.001

CI, confidence interval; ECOG, Eastern Cooperative Oncology Group; HR, hazard ratio; NOS, not otherwise specified.

## Discussion

In this study, we compared phenotype, management, and outcomes of CCA in distinct cohorts of patients with PSC, IBD without PSC, and a control cohort without PSC or IBD. PSC or IBD, or the distinct demographic features of these cohorts, did not impact overall disease state at presentation or prognosis once CCA had developed.

### *Demographics and features of* *IBD*

Patients with PSC or IBD in our cohort were younger. PSC patients had a male predominance, higher rate of UC as opposed to Crohn's compared to the IBD cohort, and the highest frequency of liver transplantation for any indication. All of this is consistent with prior studies.^6^, ^20^ Duration of IBD or history of resection for IBD did not influence survival. Although our study was not designed to assess these particular interactions, it should be noted that these features have been noted to be associated with the development of cholangiocarcinoma^10^ and survival^21^ in patients with PSC‐IBD.

There are also studies that suggest an association of IBD with CCA, independent of PSC,^11^, ^12^, ^13^ but a definitive link has not yet been found. A simple estimation, using Canadian prevalence numbers and total numbers from our cohorts, suggests that the prevalence of CCA is higher in patients with IBD compared to the general population. Of course, this is a rough estimate and should be used more so as a hypothesis‐generating exploration at this time rather than definitive data. It is possible that IBD‐only patients in our study had undiagnosed, subclinical PSC. A large population study revealed that patients with long‐standing IBD had a threefold higher prevalence of PSC on magnetic resonance cholangiopancreatography (MRCP) imaging than would have been detected via symptomatic assessment.^22^ Furthermore, two studies have shown that biochemical (alkaline phosphatase) levels may not increase over time in those with subclinical PSC.^22^, ^23^ As such, these patients may never undergo cross‐sectional screening as a lack of biochemical elevation would classically signal to physicians that PSC has been ruled out. Furthermore, in Canada, there are no formal screening or surveillance protocols to assess for PSC or CCA in patients with IBD, which would reinforce such trends in our study population. The debate as to whether stand‐alone IBD is associated with CCA could be settled if such findings of subclinical PSC continue to be demonstrated in further studies.

### *Cholangiocarcinoma presentation*

This disease is most often presented at overall stage 4 irrespective of cohort. Perihilar tumors presented very frequently at stage 3. PSC patients presented with perihilar tumors and, at stage 3, presented more frequently than the control cohort. PSC is thought to be most closely associated with pCCA as opposed to other CCA subtypes.^24^ Late stage at presentation of CCA is common and reflects a relative lack of symptoms and difficulty of diagnosis. Diagnosis and staging of CCA (particularly pCCA) remains challenging due to its early asymptomatic nature, difficult‐to‐access anatomical location, and inflammatory epithelial alterations that arise in the presence of PSC.^25^ There are data demonstrating that regular surveillance using cross‐sectional imaging results in earlier diagnosis and improved survival.^8^ The lack of formal screening or surveillance protocols for CCA in Canada likely explains the late stage at presentation we observed. An improved understanding of CCA in PSC may help in designing more sophisticated surveillance strategies with newer technologies such as intraductal ultrasound or liquid biopsy.^25^

### *Survival and therapy*

OS was not different between cohorts. As expected, survival decreased with increasing disease stage and worsening ECOG performance status. ECOG at the time of CCA diagnosis also did not differ between cohorts, suggesting good functional status of the older control cohort. Of note, although increasing age predicted worse survival in the univariate analysis, it was not associated with worse survival in the multivariate analysis. This suggests that prognosis is dismal once CCA develops, even in younger patients such as those with PSC. Patients with intrahepatic tumors, without evidence of perihilar or distal extension, had improved survival in the control and PSC cohorts. These patients did not have higher rates of resection, but disease stage was not known for most of these patients (>72%). Many of these tumors could have been of earlier stage, which would explain the improved survival. Intrahepatic tumors limited to the liver parenchyma are more likely to display mass‐forming behavior.^26^ It is possible these mass lesions may be detected at an earlier stage of disease via noninvasive means more frequently than other subtypes of CCA, leading to earlier therapy. However, this is currently speculation, and this would need further rigorous assessment.

There was no difference in rate of surgical resection by cohort, and resection with curative intent improved survival irrespective of patient cohort. Selecting appropriate resection candidates is challenging; many patients are found to have occult metastatic disease at time of resection, and some are even found to have benign disease after resection.^27^ This, again, arises from difficulty in the early assessment of CCA, and improvements in disease detection would likely further increase resection rates and overall survival. Unresectable patients may be better suited for OLT, and outcomes may be comparable in appropriate patient groups.^28^ Only nine patients in our cohort underwent OLT, but indications were not solely for CCA, and at least five of these patients were lost to follow‐up. As such, we could not directly compare OLT to resection in our cohort. Regardless, using data from the literature, one could argue that patients with PSC may simply benefit from early transplantation before CCA ever develops. Patients with PSC or IBD in our study also had very low rates of chemotherapy utilization. The impact of neoadjuvant or adjuvant chemotherapy appears to depend on CCA subtype, and there are several studies that present conflicting results with regard to survival.^27^, ^29^, ^30^ Regardless, most societies and guidelines recommend the use of chemotherapy in resectable, unresectable, and metastatic disease.^31^ Our patients with PSC or IBD were younger and one could argue that younger patients may forego chemotherapy, but in other malignancies, such as colorectal, there is actually a trend toward overutilization of chemotherapy in young patients.^32^ Existing PSC may have rendered a higher proportion of these patients with limited liver function relative to those without PSC, which would be a contraindication to chemotherapy.^33^ However, this still does not explain why those with IBD alone would have limited chemotherapy use, and we cannot fully explain this at this time. We are not aware of any literature that has noted any of these specific trends. These findings warrant further investigation to mitigate factors that may preclude chemotherapy use and determine if increased use could improve survival in these cohorts.

In conclusion, our study has several limitations. The study period spans 10 years, and management patterns of cholangiocarcinoma may have changed over this time. The data lack some granularity regarding specific features of IBD. We could not determine whether chemotherapy had been given in an adjuvant or palliative manner. Finally, there was a lack of data regarding indications and outcomes surrounding liver transplantation in these patients.

Regardless, this study has demonstrated several important findings. Despite the younger age of patients with PSC or IBD, survival was dismal once CCA developed, and most tumors presented at a late stage. Survival also appeared to be dependent on tumor location; those with isolated intrahepatic tumors did better. Our data also suggest that patients with IBD alone have an increased risk of developing CCA in comparison to controls. Finally, patients with earlier‐stage tumors amenable to surgical resection had improved outcomes. Overall, these data suggest that current screening and management protocols are not adequate for high‐risk populations such as those with PSC. In the future, development of clinical protocols able to detect and survey CCA at an early stage will be of greatest benefit for these populations.

## References

[1] Blechacz B . Cholangiocarcinoma: current knowledge and new developments. Gut Liver. 2017; 11: 13–26. 27928095 10.5009/gnl15568 PMC5221857

[2] Rizvi S , Khan SA , Hallemeier CL , Kelley RK , Gores GJ . Cholangiocarcinoma—evolving concepts and therapeutic strategies. Nat. Rev. Clin. Oncol. 2018; 15: 95–111. 28994423 10.1038/nrclinonc.2017.157 PMC5819599

[3] Endo I , Gonen M , Yopp AC *et al* Intrahepatic cholangiocarcinoma: rising frequency, improved survival, and determinants of outcome after resection. Ann. Surg. 2008; 248: 84–96. 18580211 10.1097/SLA.0b013e318176c4d3

[4] Tyson GL , El‐Serag HB . Risk factors for cholangiocarcinoma. Hepatology. 2011; 54: 173–84. 21488076 10.1002/hep.24351 PMC3125451

[5] Lazaridis KN , LaRusso NF . Primary sclerosing cholangitis. N. Engl. J. Med. 2016; 375: 1161–70. 27653566 10.1056/NEJMra1506330 PMC5553912

[6] Fung BM , Tabibian JH . Cholangiocarcinoma in patients with primary sclerosing cholangitis. Curr. Opin. Gastroenterol. 2020; 36: 77–84. 31850928 10.1097/MOG.0000000000000616

[7] Goeppert B , Folseraas T , Roessler S *et al* Genomic characterization of cholangiocarcinoma in primary sclerosing cholangitis reveals novel therapeutic opportunities. Hepatology. 2020 10.1002/hep.31110 31925805

[8] Ali AH , Tabibian JH , Nasser‐Ghodsi N *et al* Surveillance for hepatobiliary cancers in patients with primary sclerosing cholangitis. Hepatology. 2018; 67: 2338–51. 29244227 10.1002/hep.29730

[9] Sørensen JØ , Nielsen OH , Andersson M *et al* Inflammatory bowel disease with primary sclerosing cholangitis: a Danish population‐based cohort study 1977‐2011. Liver Int. 2018; 38: 532–41. 28796371 10.1111/liv.13548

[10] Gulamhusein AF , Eaton JE , Tabibian JH , Atkinson EJ , Juran BD , Lazaridis KN . Duration of inflammatory bowel disease is associated with increased risk of cholangiocarcinoma in patients with primary sclerosing cholangitis and IBD. Am. J. Gastroenterol. 2016; 111: 705–11. 27002801 10.1038/ajg.2016.55 PMC5027894

[11] Huai J‐P , Ding J , Ye X‐H , Chen Y‐P . Inflammatory bowel disease and risk of cholangiocarcinoma: evidence from a meta‐analysis of population‐based studies. Asian Pac. J. Cancer Prev. 2014; 15: 3477–82. 24870743 10.7314/apjcp.2014.15.8.3477

[12] Welzel TM , Mellemkjaer L , Gloria G *et al* Risk factors for intrahepatic cholangiocarcinoma in a low‐risk population: a nationwide case‐control study. Int. J. Cancer. 2007; 120: 638–41. 17109384 10.1002/ijc.22283

[13] Shaib YH , El‐Serag HB , Davila JA , Morgan R , McGlynn KA . Risk factors of intrahepatic cholangiocarcinoma in the United States: a case‐control study. Gastroenterology. 2005; 128: 620–6. 15765398 10.1053/j.gastro.2004.12.048

[14] Björnsson E , Angulo P . Cholangiocarcinoma in young individuals with and without primary sclerosing cholangitis. Am. J. Gastroenterol. 2007; 102: 1677–82. 17433022 10.1111/j.1572-0241.2007.01220.x

[15] Chun YS , Pawlik TM , Vauthey J‐N . 8th Edition of the AJCC cancer staging manual: pancreas and hepatobiliary cancers. Ann. Surg. Oncol. 2018; 25: 845–7. 28752469 10.1245/s10434-017-6025-x

[16] Keedy AW , Breiman RS , Webb EM , Roberts JP , Coakley FV , Yeh BM . Determinants of second‐order bile duct visualization at CT cholangiography in potential living liver donors. Am. J. Roentgenol. 2013; 200: 1028–33. 23617485 10.2214/AJR.11.8364 PMC5308084

[17] Rose KL , Sherman PM , Cooke‐Lauder J *et al* The impact of inflammatory bowel disease in Canada 2018: IBD research landscape in Canada. J. Can. Assoc. Gastroenterol. 2019; 2: S81–91. 31294388 10.1093/jcag/gwy057 PMC6512242

[18] Barner‐Rasmussen N , Pukkala E , Jussila A , Färkkilä M . Epidemiology, risk of malignancy and patient survival in primary sclerosing cholangitis: a population‐based study in Finland. Scand. J. Gastroenterol. 2020; 55: 74–81. 31902255 10.1080/00365521.2019.1707277

[19] Liang H , Manne S , Shick J , Lissoos T , Dolin P . Incidence, prevalence, and natural history of primary sclerosing cholangitis in the United Kingdom. Medicine. 2017; 96: e7116. 28614231 10.1097/MD.0000000000007116 PMC5478316

[20] Ricciuto A , Kamath BM , Griffiths AM . The IBD and PSC phenotypes of PSC‐IBD. Curr. Gastroenterol. Rep. 2018; 20: 16. 29594739 10.1007/s11894-018-0620-2

[21] Nordenvall C , Olén O , Nilsson PJ *et al* Colectomy prior to diagnosis of primary sclerosing cholangitis is associated with improved prognosis in a nationwide cohort study of 2594 PSC‐IBD patients. Aliment. Pharmacol. Ther. 2018; 47: 238–45. 29064110 10.1111/apt.14393

[22] Lunder AK , Hov JR , Borthne A *et al* Prevalence of sclerosing cholangitis detected by magnetic resonance cholangiography in patients with long‐term inflammatory bowel disease. Gastroenterology. 2016; 151: 660.e4–9.e4. 27342213 10.1053/j.gastro.2016.06.021

[23] Culver E *et al* Prevalence and long‐term outcome of sub‐clinical primary sclerosing cholangitis in patients with ulcerative colitis. SSRN Electron. J. 2020 Available at SSRN: 10.2139/ssrn.3514713 32841490

[24] Banales JM , Cardinale V , Carpino G *et al* Expert consensus document: cholangiocarcinoma: current knowledge and future perspectives consensus statement from the European Network for the Study of Cholangiocarcinoma (ENS‐CCA). Nat. Rev. Gastroenterol. Hepatol. 2016; 13: 261–80. 27095655 10.1038/nrgastro.2016.51

[25] Rizvi S , Eaton J , Yang JD , Chandrasekhara V , Gores GJ . Emerging technologies for the diagnosis of perihilar cholangiocarcinoma. Semin. Liver Dis. 2018; 38: 160–9. 29871021 10.1055/s-0038-1655775 PMC6463495

[26] Doherty B , Nambudiri VE , Palmer WC . Update on the diagnosis and treatment of cholangiocarcinoma. Curr. Gastroenterol. Rep. 2017; 19: 2. 28110453 10.1007/s11894-017-0542-4

[27] Cillo U , Fondevila C , Donadon M *et al* Surgery for cholangiocarcinoma. Liver Int. 2019; 39: 143–55. 30843343 10.1111/liv.14089 PMC6563077

[28] Goldaracena N , Gorgen A , Sapisochin G . Current status of liver transplantation for cholangiocarcinoma. Liver Transpl. 2018; 24: 294–303. 29024405 10.1002/lt.24955

[29] Schweitzer N , Weber T , Kirstein MM *et al* The effect of adjuvant chemotherapy in patients with intrahepatic cholangiocarcinoma: a matched pair analysis. J. Cancer Res. Clin. Oncol. 2017; 143: 1347–55. 28314929 10.1007/s00432-017-2392-8 PMC11819035

[30] Rangarajan K , Simmons G , Manas D , Malik H , Hamady ZZ . Systemic adjuvant chemotherapy for cholangiocarcinoma surgery: a systematic review and meta‐analysis. Eur. J. Surg. Oncol. 2020; 46: 684–93. 31761507 10.1016/j.ejso.2019.11.499

[31] Ramírez‐Merino N , Aix SP , Cortés‐Funes H . Chemotherapy for cholangiocarcinoma: an update. World J. Gastrointest. Oncol. 2013; 5: 171–6. 23919111 10.4251/wjgo.v5.i7.171 PMC3731530

[32] Manjelievskaia J , Brown D , McGlynn KA , Anderson W , Shriver CD , Zhu K . Chemotherapy use and survival among young and middle‐aged patients with colon cancer. JAMA Surg. 2017; 152: 452–9. 28122072 10.1001/jamasurg.2016.5050 PMC5806125

[33] Grimsrud MM , Folseraas T . Pathogenesis, diagnosis and treatment of premalignant and malignant stages of cholangiocarcinoma in primary sclerosing cholangitis. Liver Int. 2019; 39: 2230–7. 31216595 10.1111/liv.14180

